# Efficacy of a referral and physical activity program for survivors of prostate cancer [ENGAGE]: Rationale and design for a cluster randomised controlled trial

**DOI:** 10.1186/1471-2407-11-237

**Published:** 2011-06-13

**Authors:** Patricia M Livingston, Jo Salmon, Kerry S Courneya, Cadeyrn J Gaskin, Melinda Craike, Mari Botti, Suzanne Broadbent, Bridie Kent

**Affiliations:** 1 Faculty of Health, Deakin University, 221 Burwood Highway, Burwood, Victoria 3125, Australia; 2 Behavioural Medicine Laboratory, Faculty of Physical Education and Recreation, E-488 Van Vliet Center, University of Alberta, Edmonton, Alberta T6G 2H9, Canada; 3 School of Sport & Exercise Science, Victoria University, PO Box 14428, Melbourne, Victoria 8001, Australia

## Abstract

**Background:**

Despite evidence that physical activity improves the health and well-being of prostate cancer survivors, many men do not engage in sufficient levels of activity. The primary aim of this study (ENGAGE) is to determine the efficacy of a referral and physical activity program among survivors of prostate cancer, in terms of increasing participation in physical activity. Secondary aims are to determine the effects of the physical activity program on psychological well-being, quality of life and objective physical functioning. The influence of individual and environmental mediators on participation in physical activity will also be determined.

**Methods/Design:**

This study is a cluster randomised controlled trial. Clinicians of prostate cancer survivors will be randomised into either the intervention or control condition. Clinicians in the intervention condition will refer eligible patients (n = 110) to participate in an exercise program, comprising 12 weeks of supervised exercise sessions and unsupervised physical activity. Clinicians allocated to the control condition will provide usual care to eligible patients (n = 110), which does not involve the recommendation of the physical activity program. Participants will be assessed at baseline, 12 weeks, 6 months, and 12 months on physical activity, quality of life, anxiety, depression, self-efficacy, outcome expectations, goals, and socio-structural factors.

**Discussion:**

The findings of this study have implications for clinicians and patients with different cancer types or other chronic health conditions. It will contribute to our understanding on the potential impact of clinicians promoting physical activity to patients and the long term health benefits of participating in physical activity programs.

**Trial registration:**

Australia and New Zealand Clinical Trials Register (ANZCTR): ACTRN12610000609055

Deakin University Human Research Ethics Approval 2011-085

## Background

Worldwide, prostate cancer is the second most common cancer in men. In 2008, 899,120 cases of prostate cancer were recorded, which represents 13.6 per cent of all new cancer cases in men. In the same year, 258,133 deaths were attributable to prostate cancer. It is predicted that the number of cases will almost double (1.7 million) by 2030 [1].

Prostate cancer is the most commonly diagnosed cancer in Australia and has the third highest mortality rate after lung and bowel cancers [2]. In 2007, 19,403 new cases of prostate cancer were diagnosed and there were 2,938 deaths. The rate of prostate cancer increases rapidly from the age of 45 years [2]. Improvements in five-year survival rates have been observed for prostate cancer, from 57% to 85% [3].

With improved survival rates, cancer survivors can derive substantial functional, physical, and psychological benefits from physical activity [4]. In men with prostate cancer, systematic review evidence suggests that physical activity has the potential to enhance health-related quality of life, muscular fitness, and physical functioning, as well as reduce fatigue [5]. Although these findings are promising, additional studies (especially randomised controlled trials in cancer types other than breast cancer) need to be conducted to substantiate the work in this area [4].

For prostate cancer survivors who have been treated with androgen deprivation therapy (ADT), there may be additional reasons to undertake physical activity. Previous research has shown that after 36 weeks of ADT prostate cancer patients decreased whole body lean mass by 2.4%, bone mineral content and density of 2.4%, serum testosterone of 93.3%, PSA levels of 98.2%, and haemoglobin levels of 8.8%, as well as increases in fat mass of 13.8% [6]. Assisting men who are receiving ADT to become physically active is, therefore, necessary to reduce the risk of obesity, osteoporosis and sarcopenia.

Many men reduce their involvement in physical activity following a diagnosis of prostate cancer [7]. Although evidence is mounting on the benefits of regular physical activity for prostate cancer survivors, the prevalence of physical activity among this group is modest and varies widely between studies [5]. To improve the uptake of physical activity among prostate cancer survivors, strategies need to be developed and tested for their effectiveness.

Research has highlighted gaps in information provision regarding the type and amount of physical activity that prostate cancer survivors should undertake [8]. A key challenge is to make such information accessible to all prostate cancer survivors. One way in which this information may be conveyed is via clinicians with referrals to accredited exercise physiologists. However, research has shown that most prostate cancer survivors do not recall receiving information from clinicians about integrating physical activity into their lives [8]. Patients have reported clinicians are the most important conveyors of information [9] and a prescription or referral to an exercise physiologist may be effective in promoting physical activity among patients.

Evidence on the efficacy of prescriptions of physical activity in general practice (GP) has been mixed [10-13]. In general, GP prescriptions of physical activity have led to a moderate increase in physical activity and fitness levels for 6 to 12 months [14]. There have been few studies, however, on the efficacy of physical activity prescriptions among people with specific chronic health conditions and where physical activity prescriptions are supplemented with other interventions, such as brief counselling [11]. Findings from this limited research have yielded less than optimal results. Overall however, the findings of previous studies are sufficiently strong to warrant further work in this area. The experience of cancer may mean that survivors more readily comply with the advice of their clinicians, which would result in higher levels of physical activity from an intervention that utilises clinician referral to an exercise physiologist in this population.

Accredited exercise physiologists are specialists who can provide prostate cancer survivors with expert advice on how to increase their physical activity levels safely. Since 2006 exercise physiologists accredited by Exercise and Sport Science Australia (ESSA) have been part of Australia's universal healthcare system (Medicare). Medicare provides access to free or subsidised treatment from medical practitioners, such as accredited exercise physiologists. Given their place in the health system, accredited exercise physiologists are well positioned to make an important contribution to increasing the health of prostate cancer survivors.

A criticism of previous randomised controlled trials that have examined physical activity in cancer survivor populations is that they do not incorporate a theoretical framework. Theoretical frameworks are necessary to guide the development and evaluation of interventions so that the mechanisms that change behaviour can be understood and replicated in future interventions [15]. There are compelling reasons for using social cognitive theory [16] to underpin a physical activity intervention for prostate cancer survivors. First, recent research conducted with (a) participants of similar ages to prostate cancer survivors who are likely to participate in this planned study [17], and (b) cancer patients [18] have shown that social cognitive theory constructs explain substantial amounts of variance in physical activity. For example, in a study of middle-aged and young-old adults, social cognitive theory constructs explained 71% of the variance in physical activity [17]. Second, social cognitive theory constructs are not only predictive of health behaviours, but provide avenues for modifying them [16,19].

The main determinants of health behaviour in social cognitive theory are self-efficacy, outcome expectations, goals, and socio-structural factors [19,20]. Self-efficacy is central to this conceptual model, and "refers to beliefs in one's capabilities to organise and execute the courses of action required to produce given attainments" (p. 3, [19]). Distinctions can be drawn between various types of self-efficacy, with task and self-regulatory (coping) self-efficacy common in the exercise and physical activity literature [21]. Task self-efficacy refers to the belief in one's ability to perform a given motor skill successfully (e.g., walking briskly for 30 minutes), whereas self-regulatory self-efficacy refers to one's ability to perform the skill under conditions that may be challenging to successful performance (e.g., inclement weather, tiredness).

Outcome expectations refer to the expected effects of one's behaviour [19]. These effects may be physical (beneficial or detrimental), social (favourable or adverse), or self-evaluative (positive or negative). Goals provide guidance to, and incentives for, behaviour. In social cognitive theory, goals are classified as being either proximal or distal [19]. Proximal goals are essentially equivalent to intentions and guide current behaviours, whereas distal goals orientate future behaviours. Socio-structural factors are facilitators and impediments to healthy behaviours. These factors may be personal or situational, or lie within the health system. Enhancing physical activity-related self-efficacy in prostate cancer survivors and assisting them to develop positive outcome expectations, set beneficial goals, and reduce perceived impediments may result in higher physical activity levels.

Our pilot work indicated that outcome expectations for prostate cancer survivors to perform physical activity reflected psychological and physical benefits that can be attained through participation, as well as from the context of activity, i.e. socio-structural factors, including attractive locations, opportunities for spending time alone, social interaction [8]. Conversely, impediments to participation included limited confidence following treatment, lack of time, co-morbidities, and age-related functional decline. Despite the benefits of physical activity, men in this study did not recall receiving advice from their clinicians about physical activity and few reported being referred to exercise professionals. Given these findings, interventions to increase physical activity in prostate cancer survivors that incorporate referrals from clinicians to exercise professionals and a well constructed theoretically tailored physical activity program may achieve positive outcomes.

### Study aims and hypotheses

The primary aim of the ENGAGE study is to compare the efficacy of a clinician referral and 12-week, supervised, exercise physiologist-led physical activity program compared to usual care (no referral to the physical activity program and typically minimal advice about physical activity) in improving the physical activity levels of prostate cancer survivors at three post-intervention time points:12 weeks, 6 months, 12 months. We hypothesise that participants in the intervention condition will be more physically active than participants in the control condition.

A secondary aim is to determine the effects of the clinician referral and physical activity program on the psychological well-being, quality of life and objective physical functioning of prostate cancer survivors. At the three post-intervention time points (12 weeks, 6 months, 12 months), we hypothesise that the clinician referral and physical activity program will improve participants' quality of life and decrease anxiety and depressive symptoms over and above changes on such measures that participants in the control condition may experience at post intervention. We will measure objective physical functioning at baseline and 12 weeks only, and hypothesise that participants in the intervention condition will improve their exercise capacity more than those in the control condition.

A further aim is to assess the impact of the intervention on main health determinants using the social cognitive theory and whether these determinants mediate behaviour change. We hypothesise that the intervention will have positive effects on self-efficacy, outcome expectations, goals, and socio-structural factors; and that self-efficacy will have a direct association with physical activity and an indirect association, mediated by outcome expectations, goals, and socio-structural factors.

## Methods

### Design

This study is a cluster randomised controlled trial (RCT) to test the efficacy of an intervention, i.e., a clinician's referral to a physical activity program, to generate a) behaviour change (i.e., increasing physical activity levels) that is sustained over time and b) improved psychological well-being, quality of life, and objective physical functioning outcomes among prostate cancer survivors. The potential influence of individual and environmental mediators on participation in physical activity will also be determined.

Clinicians who agree to be involved in the study will be randomized into the intervention or control condition prior to the commencement of the study via computer-generated table of random numbers. Blinding of the clinician is not possible in this study, as one of our aims is to assess if the referral to the physical activity program by clinicians' influences the uptake of the physical activity program.

Our decision to undertake a cluster RCT, randomised by clinician with no cross over, was one based on the pragmatics of our situation. Our previous experience in this field suggests that it is difficult to gain support from busy clinicians if they were required to switch between both experimental conditions in the recruitment of patients [22]. Therefore, clinicians will be randomised into one of the two conditions and patients of each clinician will be regarded as separate clusters. There are likely to be up to twelve clinicians involved in this study (three clinicians from each of the three health services). Although we anticipate the effect of variation in the health services and in clinician physical activity recommendations on the key outcomes of this research to be very small, possible differences need to be taken into account through the use of clustering [23].

### Participants

Eligible patients will be adult males who 1) have completed active treatment for prostate cancer within the previous three-12 months. Participants currently on hormone treatment will be eligible to participate; 2) were treated with curative intent, representing stages I, II, or III; and (3) have the ability to complete surveys in the English language. Patients will be excluded from this study if they have musculoskeletal, cardiovascular, or neurological disorders that could limit them from exercising. The patients' treating clinicians will assess their eligibility for the study.

The sample size for this study was calculated based on the primary research question; number of minutes of moderate or strenuous physical activity in a typical week in past month as measured by an adapted version of the Leisure Time Exercise Questionnaire at the completion of the 12 week intervention using the following assumptions: (1) a medium-sized effect will be found, (2) α will be set at .05, (3) power will be set at .80, (4) the attrition rate will be 10% at 12 weeks, and (5) the design effect will be 1.084. The anticipated effect size was judged from a similar project [24], where a medium-sized effect was found for the outcome of a physical activity intervention (*d *= 0.484), where the intervention group increase their total weekly minutes of physical activity by 59.3 minutes (SD = 126.6).

Physical activity levels will be measured at 12 weeks to answer the primary research question. An attrition rate of 10% is expected, based on our previous work in this area [22]. A design effect of 1.084 was included to account for clustering (ICC = .005, *m¯¬ *= 16.83). The ICC was estimated from a recently completed study investigating recruitment of newly diagnosed prostate and colorectal cancer patients (clustered by specialist) [22] and from published commentary in which the authors suggested that ICC rates of less than 0.01 have been found for practitioner prescribing rates [23]. The average size of each cluster (*m¯*) was calculated from an estimation of the number of participants needed in this study before the application of the design effect (*n *= 202) and the number of clinicians (clusters) involved in this research (*n *= 12). Based on these assumptions, 220 participants will need to be recruited.

### Physical Activity Program

Participants will initially undertake a one-on-one session with the exercise physiologist, which will involve a discussion of each participant's beliefs in his or her ability to be physically active, physical activity preferences, outcome expectations, goals, and strategies for using facilitators and overcoming barriers to performing physical activity. The topics for this discussion are based on the concepts (previously identified) within social cognitive theory that are particularly relevant to health behaviour [19,20]. This is to increase long-term adherence to physical activity.

Emphasis will be placed on exercise physiologists regularly counselling participants throughout the structured physical activity sessions. The counselling topics will be based on the constructs of social cognitive theory. Exercise physiologists involved in this study will be trained in the application of social cognitive theory to physical activity uptake and adherence for survivors of prostate cancer. Exercise physiologists will be provided with checklists to ensure that they are regularly discussing key factors, including setting goals, aligning activity with outcome expectations, overcoming barriers and drawing on facilitators.

Based on previous work with prostate cancer patients [25], participants will undertake a 12-week program that will include supervised physical activity sessions and unsupervised physical activity. During the supervised sessions, several participants may be exercising at the same time. As participants will enter and leave the program at varying times and will have different weekly schedules, no attempt will be made to allocate participants to groups that will train in 12-week blocks. The physical activity program will be tailored to suit the ability of each participant. Exercise physiologists will lead two supervised sessions per week and will advise participants on what physical activity to undertake during their one non-supervised session per week.

Each supervised session will last approximately 50 minutes and participants will be encouraged to socialise following the sessions. The goal of the exercise training is to achieve 150 minutes per week of moderate to strenuous physical activity. Following the intervention, participants will be provided with a physical activity program by the exercise physiologist. The program will be based on the preferences of the participant, in terms of setting and type of exercise. The main goal of this training will be to maintain 150 minutes per week of moderate to strenuous physical activity [26].

Supervised and unsupervised physical activity has been included as both offer unique advantages. Supervised programs have value because participants can be directly observed (which may decrease risk of injury and also improve adherence to physical activity) and unsupervised programs are beneficial because of their potential to increase long term adoption and maintenance of physical activity, especially if participants find cost effective ways to incorporate physical activity into their daily routine [27]. Combining these approaches means that participants receive guidance on physical activity techniques and principles; have regular contact with, and encouragement from, the exercise physiologists and other participants; and have opportunities to explore ways of integrating physical activity into their lives.

The content of the active exercise program was informed by Exercise Guidelines for Cancer Survivors of the American College of Sports Medicine [28] and the Australian Association for Exercise and Sport Science [29], now known as Exercise and Sports Science Australia. The general program, which will be individualised for each participant, will include three sessions per week (two supervised and one unsupervised). Each session will involve aerobic exercise, progressive resistance training, balance, and flexibility exercises. The aerobic exercise will include different modes of training (e.g., walking, jogging, cycling, cross training, rowing), for 20 minutes, at an intensity of 40% to 70% of predicted heart maximum rates or 8 to 13 on the 15-point Borg scale of perceived exertion [30,31]. Progressive resistance training exercises (two to four compound exercises for lower and upper body, one core strength exercise) will be programmed using Thera-Bands and progressing to machines and free weights. One set of 8 to 12 repetitions of each exercise will be performed in the initial session, progressing to two sets of 8 to 12 repetitions of each exercise in subsequent sessions, depending on the tolerance for exercise of each participant. In the unsupervised sessions, body weight and Thera-band exercises will be prescribed for the progressive resistance training component of the program. Balance exercises will include the one legged stand, tandem stand and walk, and one legged semi squats. Flexibility exercises will be prescribed for every major muscle group.

### Measures

Table 1 provides a summary of the constructs and their measurement for this study.

**Table 1:** Constructs and Measures

Construct	Measure
**Primary Outcome**	
Participation in Physical Activity	Self-report using an adapted version of Godin and Shephard's 'Leisure Time Exercise Questionnaire' [32]
**Secondary Outcomes**	
Objective Physical Functioning	Resting heart rate, blood pressure, limb girth circumferences, 6 minute walk, sit to stand (30 seconds), 1 repetition maximum strength test, and Apley's shoulder test [43].
Accelerometer measure	The Actigraph GT3X accelerometer is a small matchbox-sized unit worn on a belt on the hip that is a valid and reliable tool for measuring physical activity among adults [45,46], and will be worn for eight consecutive days, before the intervention commences and during the final week of the intervention.
Quality of Life	European Organization for Research and Treatment of Cancer core quality of life questionnaire (EORTC QLQ-C30, version 3 [35]) and the prostate tumour-specific module (EORTC QLQ-PR25 [36]).
Anxiety	Memorial Anxiety Scale for Prostate Cancer (MAX-PC) [37]
Depression	Centre for Epidemiological Studies Depression Inventory (CES-D) [40].
**Mediators**	
Task Self-Efficacy	Participants rate the certainty with which they believe they can perform three physical tasks. For each task, they rate the certainty with which they believe they can perform these tasks for three time durations: 10, 20, and 30 minutes. Confidence will be measured on an 11-point Likert scale, from 0 (not at all confident) to 100 (extremely confident). Measure based on exercise and cancer research in which task self-efficacy was measured [48].
Barrier Self-Efficacy	Participants rate the certainty with which they believe they can perform exercise when faced with specific barriers. Items (barriers) from Bandura's [47] self-efficacy to regulate exercise instrument, with supplementary items from research on barriers to physical activity for prostate cancer survivors [8] and research on barrier self efficacy and cancer survivors [48]. The 11-point Likert scale used in the measurement of task self-efficacy will also be used to assess barrier self-efficacy.
Outcome Expectations	Multidimensional Outcome Expectations for Exercise Scale (MOEES) [49].
Goals	Participants indicate the number of days per week they intend to perform at least 30 minutes of exercise at a light, moderate or strenuous intensity now and in 12 weeks. Items adapted from previous research using social cognitive theory constructs [51].
Socio-Cultural Factors (factors facilitating or impeding participation in physical activity)	Participants asked perceptions of frequency with which each facilitator and barrier occurs using a 5-point-Likert scale anchored by 0 (never) and 4 (always). Measure based on guidelines for the development of scales to assess barriers to physical activity for cancer patients [52] and content (i.e., facilitators and impediments) drawn from our prior work with prostate cancer survivors [8].

### Primary Outcome

Our primary outcome is the number of minutes of moderate-and strenuous physical activity per week as measured by an adapted version of the Leisure Time Exercise Questionnaire [32]. In this questionnaire, participants are asked to report the average weekly frequency and duration with which they engaged in strenuous, moderate, and light exercise over the past month. Consistent with a change made to the questionnaire in a study of colorectal cancer survivors [33], participants were asked to report the average duration of time they spent exercising at each intensity, in addition to the frequency. In addition, some of the physical activities were deleted from the instrument, because they were not common activities in Australia (e.g., horseshoes, snowmobiling). The validity and reliability of the Leisure Time Exercise Questionnaire compares favourably with other physical activity questionnaires [34].

### Secondary Outcome

The secondary outcome is the impact of the physical activity program on the psychological well-being, quality of life, and objective physical functioning of prostate cancer survivors.

### Quality of life

Quality of life will be measured using a cancer-specific instrument: the European Organization for Research and Treatment of Cancer core quality of life questionnaire (EORTC QLQ-C30, version 3 [35]) and the prostate tumour-specific module (EORTC QLQ-PR25 [36]). The EORTC QLQ-C30 comprises both multi-item subscales and single items, including five functional subscales, three symptom subscales, a global health status subscale, and six single items. The EORTC QLQ-PR25 is a 25-item questionnaire designed for use among patients with localised prostate cancer, which has four subscales: urinary symptoms, bowel symptoms, treatment-related symptoms, and sexual functioning. The EORTC QLQ-C30 has convergent and discriminant validity, is responsive to change over time, and has adequate internal consistency reliability [35]. The EORTC QLQ-PR25 also has convergent and discriminant validity, and has adequate internal consistency reliability [36].

### Anxiety

Anxiety related to prostate cancer will be measured with the Memorial Anxiety Scale for Prostate Cancer (MAX-PC) [37]. This instrument has 18 items that represent three subscales: prostate cancer anxiety, prostate specific antigen anxiety, and fear of recurrence. The scale has concurrent validity with established anxiety measures (e.g., the anxiety subscale of the Hospital Anxiety and Depression Scale [38]) and discriminant validity, as well as sound internal consistency and test-retest reliability [37,39]. The MAX-PC appears to be a more sensitive measure of cancer-related changes in anxiety than comparable measures [39].

### Depression

Symptoms of depression will be assessed using the Centre for Epidemiological Studies Depression Inventory (CES-D) [40]. This instrument has 20 items representing symptoms associated with depression. The scale has strong concurrent validity, with clinical and self-report criteria, and sound construct validity [40]. The CES-D has high internal consistency and acceptable test-retest reliability. The authors of two recent reviews of depression and emotional distress measures for use with cancer patients strongly recommend the use of the CES-D as a measure of depression [41,42].

### Objective Physical Functioning

Objective physical functioning will be assessed using standard measures: resting heart rate, blood pressure, limb girth circumferences, 6 minute walk, sit to stand (30 seconds), 1 repetition maximum strength test, and Apley's shoulder test [43]. These standard tests are simple, non-invasive, can be conducted with minimal equipment and space, and have been validated for use with older people and chronic disease groups [43].

### Accelerometer Measures

Accelerometers will be worn by participants to quantify physical activity objectively. The Actigraph GT3X accelerometer is a small matchbox-sized unit worn on a belt on the hip that is a valid and reliable tool for measuring physical activity among adults [44,45], and will be worn for seven consecutive days. Movement count thresholds based on established thresholds [45] will be used to calculate the average time participants spent in moderate-to vigorous-intensity physical activity. A specially designed macro will be used to manage the data and for examining physical activity outside of work hours for employed individuals (data are recorded in 'real time' and are extractable for user-specified time periods). The whole day will also be analysed to examine potential compensation as a result of engaging in the physical activity program.

All study participants will be asked to wear an accelerometer for two 7-day periods. For participants in the intervention condition, this will be in the week between their fitness assessment session and commencement of the 12 week physical activity program, and the week following completion of the exercise program (week 13). For participants in the control condition, the two time periods will be equivalent.

Participants will be asked to wear the accelerometer on a belt around their waist over their right hip for a seven day period, except when they are sleeping, bathing, or swimming. Accelerometers will be initialized to begin data collection at 5:00 am the day after they are received by participants (giving seven complete days of recorded activity), and the epoch of integration is set at 30 seconds. As the monitor records even slight motion as a nonzero count, a sustained 20-minute period of zero counts is considered a non-wearing period, and missing data will be imputed using the Expectation Maximization (EM) algorithm as described previously [46].

Participants who are unable to provide at least three days with a minimum of six hours of data will be excluded from this analysis as the accuracy of imputation is too imprecise. Accelerometer counts will be summarised into time spent in each of four activity intensities with thresholds for sedentary, light, moderate, and vigorous activity intensity ranges of < 100, 101-1951, 1952-5724, ≥ 5725 counts min^-1 ^respectively [45]. In addition, counts ≥ 1952/min will be converted to their metabolic equivalent (MET; multiples of resting metabolic rate in kcal kg h-1) and summed to create the total "intensity weighted" minutes (i.e., MET-minutes) of Moderate-Vigorous Physical Activity (MVPA) [45,46]

### Mediators

The influence of individual and environmental mediators on participation in physical activity will be determined by the following measures.

### Self-efficacy

Both task and barrier (self-regulatory) self-efficacy will be measured using the methods Bandura [19,47] has proposed. Task self-efficacy will be assessed through asking participants to rate how confident they are that they can perform three physical tasks: walking fast (light perspiration), running, fast swimming or cycling hard, and doing exercises with weights. For each task, they will be asked the confidence with which they believe they can perform these tasks for three time durations: 10, 20, and 30 minutes. Confidence will be measured on an 11-point Likert scale, which is anchored by 0 (not at all confident) and 100 (extremely confident). Similar items have been included in previous physical activity and cancer research in which task self-efficacy was measured [48].

Barrier self efficacy will be assessed through asking participants to rate how confident they are that they can perform physical activity when faced with specific barriers. The items (barriers) came from Bandura's [47] self-efficacy to regulate exercise instrument, with supplementary items from research of physical activity for prostate cancer survivors [8] and barrier self efficacy and cancer patients [48]. The 11-point Likert scale used in the measurement of task self-efficacy will also be used to assess barrier self-efficacy.

Outcomes will be measured using the Multidimensional Outcome Expectations for Exercise Scale (MOEES) [49], which was designed for older adults. The MOESS has 15 items that represent three sub-scales: physical, social, and self-evaluative outcome expectations. Participants respond to each item on a 5-point Likert scale from 1 (strongly disagree) to 5 (strongly agree). The instrument's construct validity has been demonstrated through confirmatory factor analysis and significant correlations with other constructs consistent with the predictions inherent in social cognitive theory [49,50]. The subscales of the MOEES have good internal consistency reliability (α = .81 to .84) [49].

### Goals

Goals will be measured using items adapted from previous physical activity research in which social cognitive theory constructs were assessed [51]. Participants will be asked to indicate the number of days per week they intend to perform at least 30 minutes of exercise at a light, moderate or strenuous intensity. At each questionnaire time point, participants will be asked their current goals and their goals in 12 weeks.

### Socio-structural factors

Socio-structural factors facilitating or impeding participation in physical activity will be measured using guidelines for the development of scales to assess barriers to physical activity for cancer patients [52]. The content (i.e., facilitators and impediments) of the scale was drawn from our prior work with prostate cancer survivors [8]. Participants will be asked their perceptions of the frequency with which each facilitator and barrier occurs using a 5-point Likert scale anchored by 0 (never) and 4 (always). These barriers and facilitators will be addressed by the exercise physiologists throughout the intervention.

### Procedures

Following ethical review and approval, we will invite clinicians from three healthcare settings to participate in the study. Clinicians will be randomly allocated to either the intervention or control conditions and trained prior to commencement of the study in the study's objectives and clinician requirements including the referral process, requested dialogue and documentation completion. Adherence to the intervention and control groups' processes, will be monitored weekly by the project manager.

Eligible patients will be identified by the health service oncology nurses prior to presentation to either the public health service outpatient clinic or private health service rooms for their follow up consultation. During clinic sessions, members of the research team will approach eligible patients and introduce the study to the patient, provide an information package and seek verbal approval to follow them up within 48 hours regarding their interest in participating in the study.

#### Intervention condition

**Figure 1:**
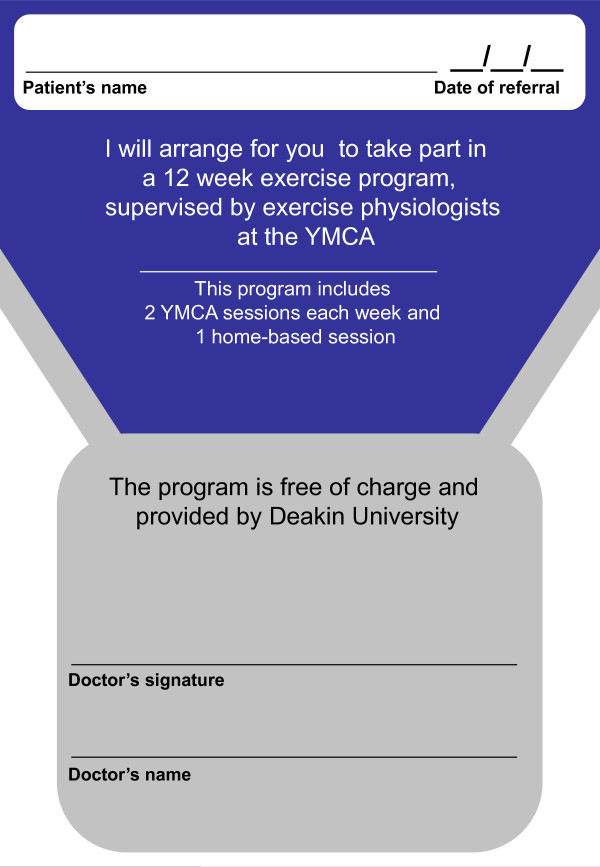
**Referral slip provided to participants by clinicians in the intervention group**.

During the consultation, clinicians randomised to the intervention arm will determine the patient's eligibility to be involved in the active exercise program and if eligible, provide each patient with a referral (Figure 1) to participate in the active exercise program and say *"I understand you have been given some information about a research project that is being conducted. You have been assigned to the exercise group. I recommend that you take part. The project manager will call you in a couple of days to see if you would like to take part in the study. Your decision to take part in the study will not affect your treatment or care in any way"*.

If the clinician does not believe that the patient is suitable for the study, they do not give the referral slip to the patient, but complete a form to indicate reasons for non-suitability (eg. co-morbidities; poor health status) and say, *"I understand you have been given some information about a research project that is being conducted. Due to your current state of health, I do not recommend that you take part in this study. I will let the researchers know that you cannot participate"*.

#### Control condition

The control condition involves participants receiving usual care from their clinicians regarding physical activity and, no referral to the active exercise program. Eligible patients will be approached by a team member to discuss the project and if the patient agrees, will be given the information package for the patient to take home and consider participating the project.

Clinicians in the control group will say, *"I understand you have been given some information about a research project that is being conducted. Although you have not been assigned to the exercise group, I recommend that you take part in the study. The project manager will call you in a couple of days to see if you would like to take part in the study. Your decision to take part in the study will not affect your treatment or care in any way"*.

If the clinician does not believe that the patient is suitable for the study, they complete a form to indicate reasons for non-suitability, and say the following:

*"I understand you have been given some information about a research project that is being conducted. Due to your current state of health, I do not recommend that you take part in this study. I will let the researchers know that you cannot participate"*.

To ensure the integrity of the trial's procedures, each of the clinicians and health service oncology nurses will undergo specific training and quality assurance assessments throughout the recruitment phase of the study.

Clinicians in both the intervention and control groups will discuss involvement in the project and provide a recommendation to participate if s/he considers it is feasible for the patient to participate. If the clinician determines the patient should not participate, s/he will complete the Reason for Non-Suitability Form and record the reason for non-participation, for example, pre-existing medical conditions, and the patient will be advised accordingly.

**Figure 2:**
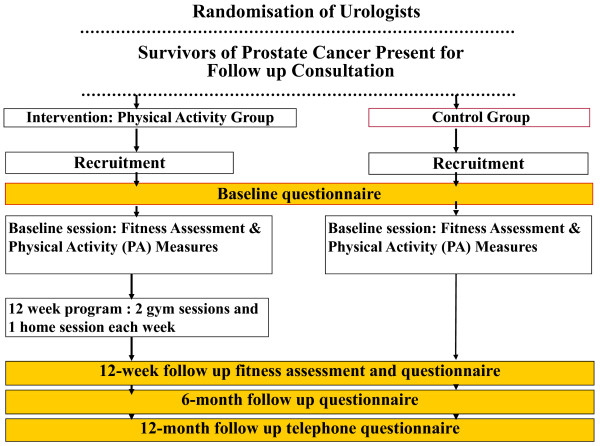
**Recruitment and data collection procedures**.

If the patient is eligible and has agreed to be followed up, the project manager will telephone them within 48 hours to determine the patient's interest in participating, answer any questions he may have and if happy to participate, request return of the signed consent form and baseline questionnaires. Once a consent form has been returned, the project manager will arrange a time for the participant's first fitness assessment. Figure 2 shows the recruitment and data collection time points for the study.

Data collection for all measures and for participants in both conditions will occur at baseline and 12 weeks (on completion of the intervention). Participants in the intervention condition will be referred to the exercise program, whereas those in the control condition will receive usual advice about exercise from their clinician. Data collection will continue for participants in both conditions and for all measures except those of objective physical functioning at 6 months and 12 months.

For those in the intervention group, over the 12 week physical activity period, three morning and three afternoon sessions between Monday and Friday will be available for participants to attend. On commencement of the program, the project manager will provide a timetable of sessions that is convenient to the participant. The exercise physiologist will contact the participant 24 hours prior to their first physical activity session each week and remind them of their session times for the week. If the participant cannot be contacted, the exercise physiologist will contact the participant the day of their physical activity session to remind them of their session time. After two failed telephone attempts and if the participant does not attend the session, the participant will be declared 'absent' for the session. The total number of sessions attended by the participants will be recorded.

### Analysis

To address the primary aim of this study, we will conduct three main tests. To assess the impact of the intervention, we will perform an analysis of covariance (ANCOVA) on the post-intervention (12-week) physical activity levels of the participants in the two conditions, with their baseline scores as a covariate [53]. To determine the sustainability of the physical activity levels at 6 and 12 months, we will perform two ANCOVA on post-intervention (6 and 12 months, respectively) physical activity levels of the participants in the two conditions, with baseline scores as covariates.

To examine the data on the first of the secondary aims (determining the effect of the clinician referral and the physical activity program on psychological well-being, quality of life, and objective physical functioning) we will use the same analytical strategies as for the primary aim (ANCOVA with baseline scores as a covariates [53]). For this analysis, the dependent variables will be the subscales of the quality of life, anxiety, and depression measures, as well as the separate measures of objective physical functioning. The immediate effects of the intervention will be assessed using the 12-week data. The sustainability of the effects of the intervention on quality of life, anxiety, and depression will be assessed at 6 and 12 months.

To address the final aim, we will use path analyses to test for possible mediation of the intervention effect on level of physical activity based on the Baron and Kenny [54] approach. We will only test the components of SCT as possible mediators if they were affected by the intervention (i.e., group assignment) and associated with follow-up participation in physical activity. This approach requires that the outcome (i.e., participation in physical activity) be regressed on the proposed mediators (i.e. SCT components) and the intervention (i.e., group assignment coded as "0"= control and "1"= intervention). Mediation is present when the proposed mediator maintains a significant relationship with the outcome whereas group assignment does not. For the path analysis, we will adjust for the same variables as in the main analyses.

For all analyses, we will employ the intention-to-treat principle for all participants with available data at post intervention and follow-up.

## Discussion

To the best of the authors' knowledge, this trial is the first to consider the efficacy of clinician referrals to a physical activity program to increase physical activity levels among cancer survivors. Although there is an expanding evidence that provides strong support for promoting physical activity among survivors of prostate cancer [5], substantial work has yet to be undertaken on how this research could be implemented into clinical practice.

This trial also has a strong emphasis on behaviour change and the mechanisms through which this change occurs. This addresses a limitation of previous research, as few randomised controlled trials of cancer survivors, or indeed other population groups, have examined the effects of a physical activity intervention on potential mediators of behaviour change and physical activity adherence using a theoretical model. In applying social cognitive theory, the intervention design addresses key constructs in social cognitive theory and examines if these factors change as a result of the intervention and if this change, in turn, explains change in physical activity participation. This investigation provides data that will assist in the design of physical activity trials that have behaviour change as a primary aim and outcome.

In the present study, we investigate one way in which the physical activity levels of prostate cancer survivors could be increased, to improve health outcomes while incorporating social and cognitive mechanisms that may contribute to desired outcomes. Findings from this research may have implications for the treatment of not only prostate cancer survivors but also other cancer survivors and those with other chronic health conditions.

## Competing interests

The authors declare that they have no competing interests.

## Authors' contributions

PML and MC developed the study concept and initiated the project. PML, MC, JS, KC, CJG, MB, SB, and BK provided significant input into the development of the protocol. Each of the authors have contributed, read and approved the final manuscript. The investigators will be involved in the steering committee, implement the protocol and/or oversee data collection.

## Pre-publication history

The pre-publication history for this paper can be accessed here:

http://www.biomedcentral.com/1471-2407/11/237/prepub
